# Long-Term Investigation of Nano-Silica Gel for Water Shut-Off in Fractured Reservoirs

**DOI:** 10.3390/gels10100651

**Published:** 2024-10-11

**Authors:** Ahmed Ali, Mustafa Al Ramadan, Murtada Saleh Aljawad

**Affiliations:** 1 College of Petroleum Engineering and Geosciences, King Fahd University of Petroleum & Minerals, Dhahran 31261, Saudi Arabia; 2 Center for Integrative Petroleum Research, King Fahd University of Petroleum & Minerals, Dhahran 31261, Saudi Arabia

**Keywords:** water shut-off, fractures, nano-silica, gel durability

## Abstract

Silicate gels have long been utilized as water shut-off agents in petroleum fields to address excessive water production. In recent years, nano-silica gel has emerged as a promising alternative to traditional silicate gels, offering potentially improved plugging performance. However, the long-term effectiveness of these gels remains uncertain, posing challenges to sustained profitability. Therefore, a comprehensive study spanning 6 months was conducted on fractured and induced channel samples treated with nano-silica gel of 75/25 wt% (silica/activator) at 200 °F. A comparative analysis was performed with samples treated using polyacrylamide/polyethyleneimine PAM/PEI gel (9/1 wt%) to compare the performance of both systems. Throughout the aging period in formation water at 167 °F, endurance tests were conducted at regular intervals, complemented by computed tomography (CT) scans to monitor any potential degradation. The results revealed nano-silica gel’s superior long-term performance in plugging fractures and channels compared to PAM/PEI gel. Even after 6 months, the nano-silica gel maintained a remarkable 100% plugging efficiency at 1000 psi, with a maximum leak-off rate of 0.088 cc/min in the mid-fractured sample and 0.027 in the induced channel sample. In comparison, PAM/PEI gel exhibited a reduction in efficiency to 99.15% in the fractured sample (5.5 cc/min maximum leak-off rate) and 99.99% in the induced channel sample (0.036 cc/min maximum leak-off rate). These findings highlight the potential of nano-silica gel as a more durable water shut-off agent for managing water production in fractures and channels.

## 1. Introduction

In the oil industry, excessive water production poses a significant challenge, with unwanted water accompanying hydrocarbons [1]. Excessive water production not only reduces operational efficiency and profitability but also raises logistical, economic, and environmental concerns [1,2]. Handling, treating, and disposing of co-produced water requires additional resources, driving operational costs up and lowering overall profitability [1]. Water shut-off techniques aim to mitigate excessive water production from reservoirs by restricting or eliminating water flow into production wells, thereby improving hydrocarbon recovery efficiency and reservoir performance. Numerous studies have highlighted the detrimental impact of produced water on well productivity. For instance, Bailey et al. [3] found that globally, three barrels of water were produced for every barrel of oil extracted, resulting in a significant volume of 75 billion barrels of co-produced water worldwide, with disposal costs estimated at USD 40 billion. Additionally, Pappaset et al. [4] revealed that the United States alone generated approximately 24.4 billion barrels of co-produced water. These statistics underscore the critical need for prompt water production treatments.

Two common solutions for mitigating water production in oil wells are mechanical and chemical methods. Mechanical methods involve placing [5,6] mechanical tools like packers and plugs in the wellbore to block water flow [7,8,9]. On the other hand, chemical methods involve placing durable materials around the wellbore or into the reservoir [1,2]. Over the last decade, various chemical systems have been developed, such as monomers, resins, and gel systems. Among these, gel treatments are considered one of the oldest and most cost-effective methods for addressing wellbore or reservoir-related issues [10,11,12,13,14,15]. These gels can be categorized into organic gels, like polymer gels, and inorganic gels, like silicate gels. Silicate gels, in particular, have been extensively used to control excessive water production. When a specially prepared silica gel mixture is injected into the wellbore or reservoir, a 3D gel structure is formed, effectively preventing water flow [16,17]. This method minimizes production disruptions, enhances reservoir sweep efficiency, and increases overall hydrocarbon recovery. Silicate gel systems offer operational advantages such as easy injection due to their low viscosity and stable performance at high temperatures. Moreover, they are cost-effective and environmentally friendly compared to polymer gels [18,19,20]. However, challenges exist, including decreasing gel strength over time, rapid gel formation, and sensitivity to certain ions in the formation [2,18,21].

In recent years, nanomaterials have emerged as promising additives in the petroleum industry, offering enhanced performance across various applications such as enhanced oil recovery, reservoir imaging, and formation evaluation [21,22,23,24]. Studies have demonstrated their effectiveness in blocking pore throats, highlighting their suitability in water shut-off treatments [25,26,27]. Research by Ali et al. [2] summarized the impact of incorporating nanomaterials like nano-silica and nano-clay into polymer gels, revealing improvements in gel properties, including gel strength and thermal stability. Furthermore, investigations into nano-silica gel highlighted its superior performance compared to larger particle sizes in silicate gel systems, making it an excellent candidate for water shut-off applications. Moreover, rheological studies have shed light on the behavior of nano-silica gel under different conditions. For instance, Boul et al. [28] found that non-spherical particles exhibit prolonged gelation times at 50 °F. Almohsin et al. [29] delved into the influence of temperature on the gelation time of nano-silica gel, observing an acceleration with increasing temperature. Additionally, their study identified higher concentrations (24% and 25%) as significantly affecting gelation time, with lower sensitivity observed at concentrations of 21% and 23%. These findings underscore the potential of nano-silica gel in water shut-off treatments and contribute valuable insights into its rheological behavior under varying conditions.

The plugging efficiency of nano-silica gel has been extensively studied in both permeable matrices and fractures. Almohsin et al. [30] evaluated this gel’s performance in a high permeable matrix, achieving successful plugging of a 400 md core sample at 300 °F over two weeks. Similarly, Karadkar et al. [31] reported 100% plugging efficiency for nano-silica gel on a 190 md Berea core sample, with no observed leak-off rate under 2500 psi differential pressure for eight hours. Additionally, Almohsin et al. [29] examined this gel’s effectiveness in plugging core samples with higher permeability (700 mD) over a 12-day endurance test, achieving an average leak-off rate of 0.0018 cc/min under continuous pumping at 4000 psi [29]. For gel performance in fractures, Ali et al. [32] investigated the performance of nano-silica gel in fractures and channels, demonstrating approximately 100% plugging efficiency with a minimal leak-off rate at 200 °F. While these studies highlight the exceptional plugging efficiency of nano-silica gel initially, none have assessed its performance over an extended period. Addressing this gap is crucial given the historical drawback of decreased gel strength over time observed in conventional silicate gel systems.

The objectives of this study encompass two primary aims: first, to evaluate the long-term durability of nano-silica gel within fractures and channels over a period of six months, and second, to conduct a comparative analysis of its performance against in situ polymer gel, specifically polyacrylamide/polyethyleneimine (PAM/PEI) gel. Prior studies have highlighted the capability of PAM/PEI gel to attain 100% plugging efficiency in Berea sandstone core samples at a temperature of 194 °F [33]. Additionally, this gel has demonstrated notable effectiveness in mitigating water flow in harsh conditions, achieving an impressive 99.8% reduction in permeability over 6 months [34]. By performing a comparative analysis between nano-silica gel and PAM/PEI gel using similar fractured sample characteristics, it is feasible to determine the superiority of one gel over the other.

## 2. Results and Discussion

### 2.1. Gel Performance on the Fractured Samples

#### 2.1.1. Experiment 1 (Nano-Silica Gel on Fractured Sample)

**Figure 1:**
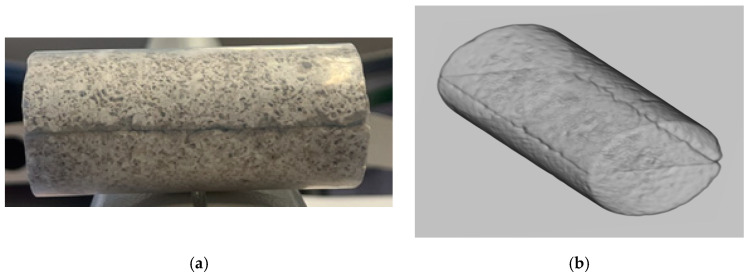
Fractured core sample images: (**a**) prepared core sample; (**b**) 3D CT scan image.

In this study, a limestone fractured sample was employed, with a fracture deliberately created in the middle section of the sample. Images of the prepared core sample and 3D CT are displayed in Figure 1. The preparation involved cutting the original sample into two similar halves, which were then reconnected using a shrinkage tube with heating. The sample’s dimensions and properties were determined using a vernier caliper and helium porosimeter, with the data summarized in Table 1. The fracture has a thickness of less than 0.1 inches along the 3-inch long sample. Notably, the fractured sample exhibited a permeability of 184.73 md, significantly higher than that of the original sample.

According to the experimental conditions (200 °F) and based on previous gelation time study [29], the prepared nano-silica gel had a concentration of 75% nano-silica and 25% activator. This concentration was chosen to provide approximately 4 h of gelation time, allowing for proper gel placement and preventing premature plugging. The brine used was composed mainly of NaCl and was used in the post-treatment stage to conduct an endurance test.

##### Gel Treatment

**Figure 2:**
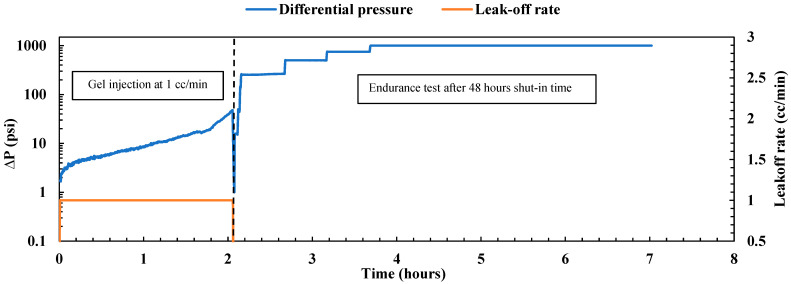
Nano-silica gel treatment on the fractured sample.

The experiment commenced under reservoir conditions at 200 °F and a confining pressure of 2000 psi, with the sample fully saturated with brine. The sample’s permeability was first evaluated by the injection of brine at four different flow rates (1, 2, 3, and 4 cc/min). The estimated average permeability was 184.73 mD, significantly exceeding the original sample’s permeability without the mid-fracture, which was 70 mD. After this evaluation, nano-silica gel was injected using a mixture of 75 wt% nano-silica and 25 wt% activator, allowing for gel formation with a gelation time of 3 to 4 h. The gel was injected at a low flow rate of 1 cc/min. Figure 2 illustrates the immediate pressure drop observed during gel injection, attributed to the increasing viscosity of the solution with temperature. Over 3.5 h, a total injected volume equivalent to 6.3 pore volumes was achieved, with a pressure drop of less than 50 psi and a gel resistance factor of 17.9 (ratio of pressure drop for the gel to pressure drop for the water), indicating satisfactory gel injectivity. Following the injection process, the system underwent cleaning and then was shut in for 48 h to allow the gel to cure. After this period, the gel plugging performance was evaluated by an endurance test conducted under various differential pressures while the leak-off rate was monitored as depicted in Figure 2. Initially, the sample was subjected to 250 psi for 30 min, followed by 500 psi for an additional 30 min, with no observed leak-off rate during either period, indicating the high plugging efficiency of the nano-silica gel. Subsequently, the pressure was raised to 750 psi and maintained for 30 min, with no droplets observed in the outlet. To assess gel durability, the differential pressure was further increased to 1000 psi and maintained for 3 h. The leak-off rate during this period remained very low at 0.054 cc/min, with a holding pressure of 4000 psi/ft, underscoring the gel’s plugging capabilities under high differential pressure. The gel effectively obstructed both the fracture path and the permeable matrix, achieving a plugging efficiency of 100%. Moreover, it exhibited a high residual resistance factor of 39,746.9 (ratio of initial permeability to final permeability), significantly reducing the sample permeability from 184.73 mD to 0.0046 mD. These results align closely with previous findings on nano-silica gel obtained by Almoshin et al. [29], highlighting the exceptional plugging capabilities of the gel.

**Figure 3:**
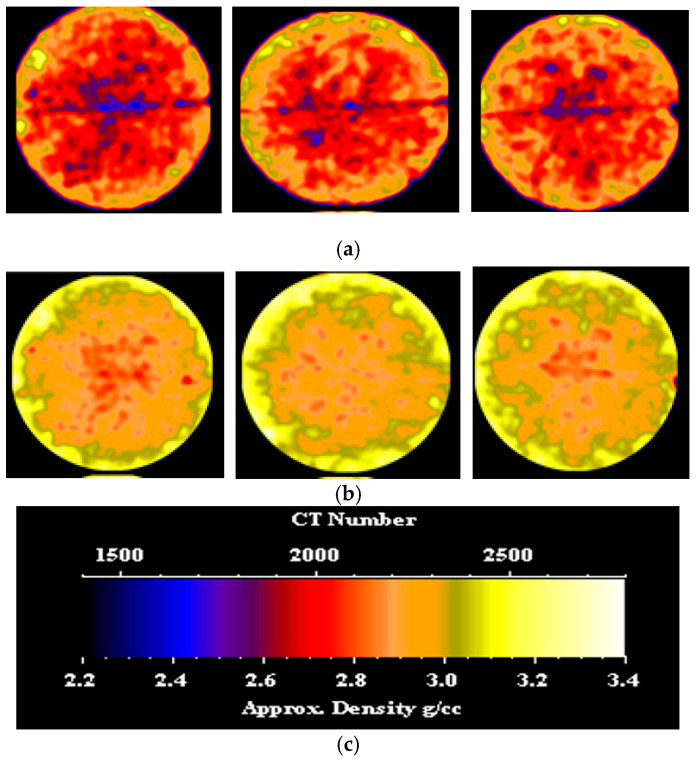
(**a**) CT scan of the fractured sample before nano-silica gel treatment; (**b**) CT scan of the fractured sample after nano-silica gel treatment; (**c**) color code scale.

CT scans were performed on the core sample, both before and after nano-silica gel treatment. Figure 3 showcases CT images depicting various slices of the dry sample and the treated sample. From the left side, the slices are located 0.5 inches, 1.7 inches, and 2.7 inches from the inlet face, respectively. It is evident that the gel induced a shift in the density distribution of the sample, resulting in a broader spread of higher density compared to the initial low-density distribution observed prior to gel application. This alteration can be attributed to the infiltration of the gel. In the section, particularly in the fracture area, a clear shift to higher density is evident, attributable to the presence of the gel. Prior to gel treatment, the average CT number of each slice was recorded at 2104.28, while post-treatment, this value increased significantly to approximately 2321.18. Such a substantial change signifies successful placement and uniform distribution of the gel along the sample length, effectively sealing the fracture path and achieving the desired plugging efficiency.

##### Gel Durability over a 6-Month Period

**Figure 4:**
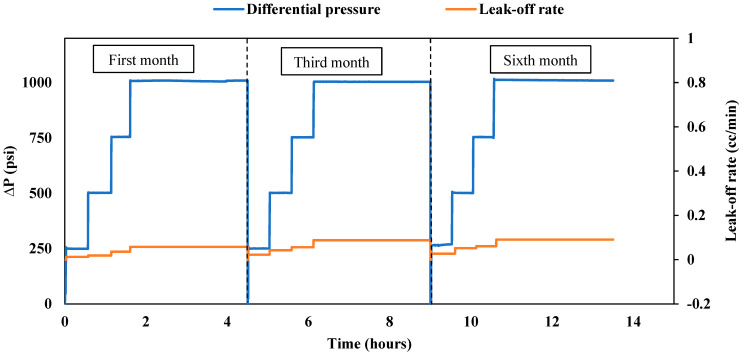
Nano-silica gel plugging performance over 6 months (fractured sample).

**First month of aging:** The treated fractured sample underwent aging within a cell filled with formation water at a temperature of 167 °F for one month. Following the aging period, an endurance test lasting 4.5 h was conducted to assess the plugging efficiency of the sample, as illustrated in Figure 4. The test commenced with a 30 min pressure hold at 250 psi, during which no leak-off rate was observed at the outlet. Subsequently, the pressure was incrementally raised to 500 psi for another 30 min and then further increased to 750 psi for an additional half-hour. During this period, the leak-off rate consistently remained below 0.036 cc/min, which closely resembled the post-treatment leak-off observed under the same differential pressure. To evaluate the durability of the gel, the pressure was maintained at a constant 1000 psi for 3 h, resulting in a reported leak-off rate of 0.058 cc/min. Remarkably, the maximum leak-off rate remained stable and nearly identical to the post-treatment leak-off rate (0.054 cc/min), affirming the gel’s durability under high differential pressure and maintaining its plugging efficiency at 100%. The consistent performance over the entire duration highlights the gel’s ability to sustain its plugging efficiency at 100%, showing minimal degradation even under significant pressure stress.

**Third month of aging:** After the initial month of endurance testing, the sample underwent further immersion in formation water at 167 °F for an additional two months. Following this period, a subsequent endurance test was carried out, involving the injection of brine under different pressure differentials, reaching a maximum of 1000 psi at 200 °F. As depicted in Figure 4, a slight uptick in the leak-off rate was observed during the third month compared to the first. Pressures of 250, 500, and 750 psi were maintained for 30 min each, with the maximum leak-off rate remaining minimal at 0.056 cc/min. To assess the gel’s endurance, the pressure was kept constant at 1000 psi for 3 h, resulting in a reported leak-off rate of 0.088 cc/min. In a comparison of this third-month leak-off rate with the post-treatment values (as depicted in Figure 2), a very slight increase was noted. Nevertheless, the leak-off rate remained extremely low, with the overall plugging efficiency of the gel staying at 100%. In a comparison of the post-treatment and third-month leak-off rates, the gel demonstrated only minimal performance degradation, with the plugging efficiency remaining at 100%. This performance reveals that, even after three months, the gel retains excellent sealing properties.

**Sixth month of aging:** After the third-month endurance test, the sample underwent an additional three months of immersion in formation water at 167 °F. The endurance test followed the same procedure, gradually increasing the differential pressure to 1000 psi at 200 °F. The performance of the gel during the sixth month is visually illustrated in Figure 4. A noticeable similarity in the leak-off rate compared to the third month was observed, indicating the gel’s stability and consistent performance. At 500 psi for 30 min, the leak-off was around 0.052 cc/min. This increased slightly to 0.061 cc/min at 750 psi. When held at 1000 psi for 3 h, the leak-off stabilized at 0.091 cc/min, similar to the rate obtained in the third month (0.088 cc/min). Nevertheless, the maximum leak-off rate obtained is exceedingly low, confirming the gel’s remarkable effectiveness in sealing the fractured sample. These findings after 6 months confirm that the nano-silica gel maintained its durability, achieving a plugging efficiency of 100%. These results highlight the gel’s ability to endure extended exposure to high temperatures and pressures, maintaining its integrity over time and making it a highly reliable sealing agent for field applications involving fractured formations.

#### 2.1.2. Experiment 2 (PAM/PEI Gel on the Fractured Sample)

For this study, a fractured sample, designed similarly to that used in experiment 1, was employed. The dimensions and properties of the sample were measured using a vernier caliper and a helium porosimeter and are outlined in Table 2. A PAM/PEI gel concentration of 9/1 wt% was selected based on a previous gelation time study [34], which provided a gelation time of over 6 h at 200 °F, making it suitable for gel placement. Notably, the fractured sample exhibited a permeability of 242.58 md, which is significantly higher than that of the original sample (70 mD).

##### Gel Treatment

**Figure 5:**
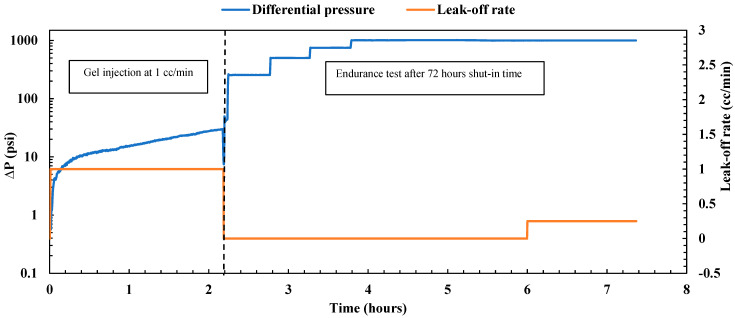
PAM/PEI gel treatment in the fractured sample.

Under reservoir conditions at 200 °F, brine was injected at four different flow rates in the saturated sample to determine the sample’s permeability, which was found to be 242.58 mD, significantly higher than the original sample’s permeability (without the fracture) of around 70 mD. Then, following the preheating of the coreflooding system, the PEM/PEI gel treatment phase began. Formulated with a concentration of 9/1 %wt with a gelation time above 6 h, the PAM/PEI gel was then injected. Figure 5 depicts the immediate pressure drop observed during the injection process. Over the course of 2 h, 7 pore volumes were injected, with the maximum pressure drop staying below 40 psi. This resulted in a gel resistance factor of 30, suggesting effective gel injectivity.

Following the injection phase, the system underwent a cleaning procedure and then remained shut in for a period of 72 h to facilitate the curing of the gel. Subsequently, an evaluation of the gel’s plugging performance was conducted through an endurance test, which involved subjecting it to varying levels of pressure differentials while monitoring the rate of leakage, as illustrated in Figure 5. Initially, the sample endured 250 psi for 30 min, followed by an additional 30 min at 500 psi, with no observable leakage during either phase, indicating the initial high efficiency of the PAM/PEI gel in plugging the fractured sample. The pressure was then increased to 750 psi and maintained for 30 min, with no detection of water droplets at the outlet. To assess the durability of the gel, the pressure was further increased to 1000 psi and sustained for 3 h. During the last hour of maintaining the pressure at 1000 psi, water droplets began to emerge, with a leak-off rate recorded at 0.25 mL/min. The gel effectively obstructed both the fracture path and the permeable matrix, achieving a remarkable plugging efficiency of 99.99%. Additionally, it demonstrated a substantial residual resistance factor of 13,380.59, leading to a significant reduction in sample permeability from 242.58 mD to 0.0181 mD. These findings validate the gel’s capacity to impede water flow in fractures.

**Figure 6:**
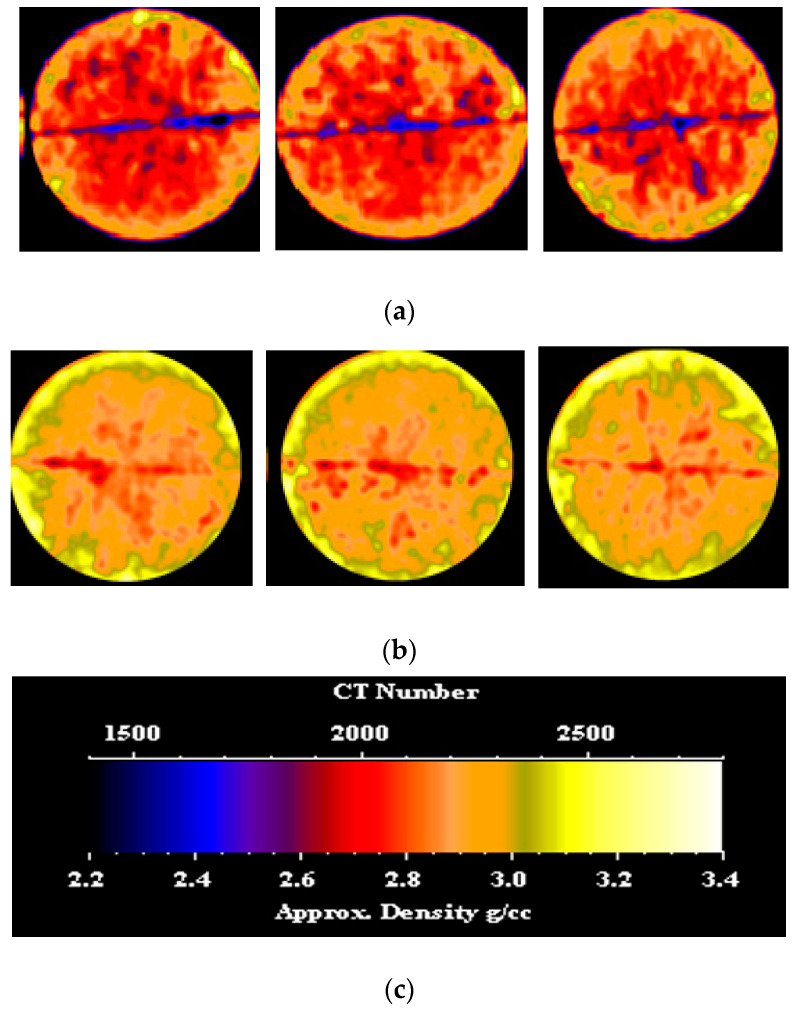
(**a**) CT scan of the fractured sample before PAM/PEI gel treatment; (**b**) CT scan of the fractured sample after PAM/PEI gel treatment; (**c**) color code scale.

CT scans were conducted on the core sample both before and after PAM/PEI gel treatment, and Figure 6 displays CT images showcasing various slices of both the dry sample and the treated sample. From the left side, the slices are located 0.5 inches, 1.7 inches, and 2.7 inches from the inlet face, respectively. Evidently, the gel induced a change in the density distribution of the sample, resulting in a broader spread of higher density compared to the initial low-density distribution observed before gel application. This alteration is attributed to gel infiltration. Notably, in the middle slice, particularly within the fracture area, a distinct shift to higher density signifies the presence of the gel. Before gel treatment, the average CT number of each slice was 2096.92, whereas post-treatment, this value substantially increased to approximately 2279.09. This significant change validates the successful placement and uniform distribution of the gel throughout the sample length, encompassing both the fracture path and the surrounding matrix.

##### Gel Durability over a 6-Month Period

**Figure 7:**
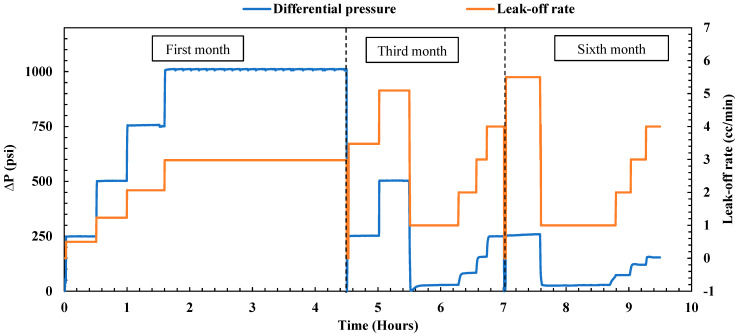
PAM/PEI gel performance over 6 months (fractured sample).

**First month of aging:** Following the initial treatment, the fractured sample underwent aging in formation water within a cell at a temperature of 167 °F for one month. Subsequently, the endurance test commenced with the injection of brine at various differential pressures, as displayed in Figure 7. Initially, a 30 min pressure hold at 250 psi revealed a noticeable leak-off rate of 0.5 cc/min, significantly higher than the post-treatment rate of zero leak-off. Gradually increasing the pressure to 500 psi for 30 min resulted in a leak-off rate of 1.233 cc/min, and further increasing the pressure to 750 psi for 30 min led to a rise in the leak-off rate to 2.067 cc/min. These observed leak-off rates were higher than the post-treatment rate, indicating an initial indication of gel degradation. To assess the gel’s durability, the pressure was maintained at a constant 1000 psi for 3 h, resulting in a reported leak-off rate of 2.98 cc/min. Consequently, the reported plugging efficiency of the PAM/PEI gel decreased to 99.88% after one month, indicating a slight degradation of the gel.

**Third month of aging:** Following the initial month of endurance testing, the sample underwent an additional two months of immersion in formation water at 167 °F. Subsequently, a subsequent endurance test was conducted at 200 °F in two stages: one using constant pressure and the second using constant flow rate. As depicted in Figure 7, a rise in the leak-off rate was observed in the third month compared to the first month. Pressures of 250 and 500 psi were sustained for 30 min each, with the leak-off rate increasing to 4.79 cc/min and then to 5.33 cc/min, respectively. These leak-off rates were considerably higher than those observed in the first month, indicating further degradation of the gel over the previous two months. Afterward, the test mode was changed to a constant flow rate to obtain accurate results about the sample permeability. Four different flow rates were applied, starting with 1 cc/min, then 2, 3, and finally 4 cc/min. The total injected volume through the treated sample was 10 pore volumes, resulting in a plugging efficiency of 99.55%. It is evident that the gel degraded after 3 months, as the plugging efficiency decreased from 100% in the post-treatment stage to 99.55%.

**Sixth month of aging:** Following the third-month endurance test, the sample underwent an additional three months of aging in formation water at 167 °F. Subsequently, the endurance test was repeated using the same procedure, initially with constant pressure and then transitioning to a constant flow rate. Figure 7 illustrates the gel’s performance after the sixth month, revealing a noticeable increase compared to the third month. At 250 psi for 30 min, the leak-off rate was measured at 7.33 cc/min, approximately 1.5 times the leak-off rate observed in the third month, indicating further degradation of the gel. Afterward, the test mode was switched to a constant flow rate, beginning with 1 cc/min and gradually increasing to 2, 3, and finally 4 cc/min. The total injected volume reached 10 pore volumes, resulting in an average permeability of 2.06 mD, which is higher than the post-treatment permeability of 0.018 mD. The plugging efficiency of the PAM/PEI gel after six months was approximately 99.15%.

The aging and endurance testing of the PAM/PEI gel over a six-month period revealed a minimal degradation in its plugging efficiency and sealing performance. Initially, after one month, the gel exhibits only minimal degradation, maintaining a high plugging efficiency of 99.88%, with relatively low leak-off rates despite exposure to differential pressures up to 1000 psi. However, as the aging process progresses to three and six months, the leak-off rates increase significantly, particularly under higher pressures, indicating that the gel’s structural integrity is being compromised over time. By the sixth month, the leak-off rates at 250 psi are approximately 1.5 times those observed in the third month, and permeability has increased from 0.018 mD post-treatment to 2.06 mD, signaling a notable reduction in the gel’s ability to maintain a tight seal. The transition to a constant flow rate test further highlights the gel’s weakening, as higher permeability levels reflect the breakdown of the gel matrix.

#### 2.1.3. Comparison of Gel Performance in the Fractured Samples

**Figure 8:**
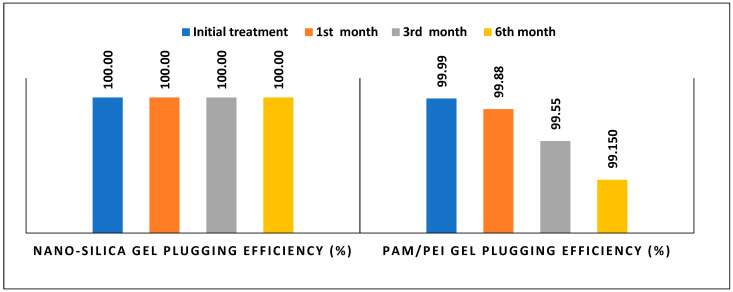
Performance comparison of nano-silica and PAM/PEI gels in the fractured sample.

Fractured samples with similar characteristics were treated by nano-silica gel and PAM/PEI gel. This assisted in constructing a valid comparison between the gel’s performance over 6 months. Figure 8 displays the plugging efficiency of both gels over 6 months at different stages. In general, it appears that over an extended period, nano-silica gel was more effective than PAM/PEI gel. Nano-silica gel maintained its durability with plugging efficiency remaining at 100%. PAM/EPI gel efficiency clearly decreased from 99.99% to 99.15% over 6 months. Additionally, The maximum leak-off rate of PAM/PEI gel at 250 psi was 5.5 cc/min, which is 5 times the leak-off rate obtained by nano-silica gel on the fractured sample. This confirms that nano-silica gel exhibits superior performance compared to PAM/PEI gel over the span of 6 months.

### 2.2. Gel Performance on the Induced Channel Samples

#### 2.2.1. Experiment 3 (Nano-Silica Gel on the Induced Channel Sample)

**Figure 9:**
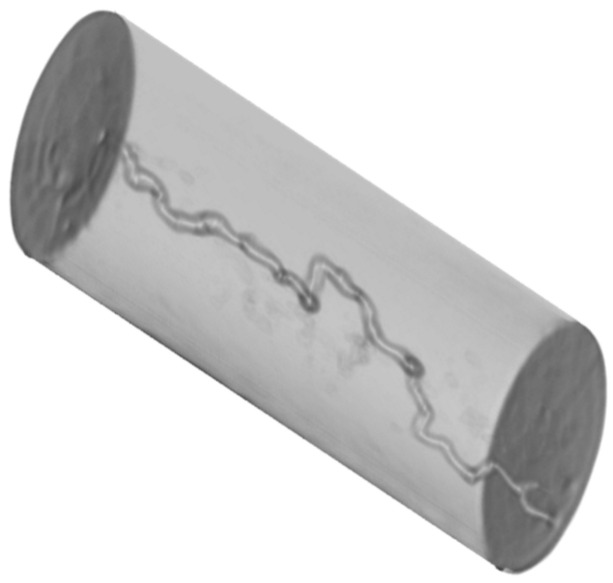
A 3D image of the induced channel sample of experiment 3.

This investigation employed a sample with a distinct characteristic—a channel (wormhole) created by hydrochloric acid (HCl). A 3D CT scan image of the sample is depicted in Figure 9. Further details regarding the dimensions and properties of the sample are outlined in Table 3. Notably, the sample exhibits a permeability of 440.6 md, significantly higher than that of the original sample (less than 50 mD).

##### Gel Treatment

**Figure 10:**
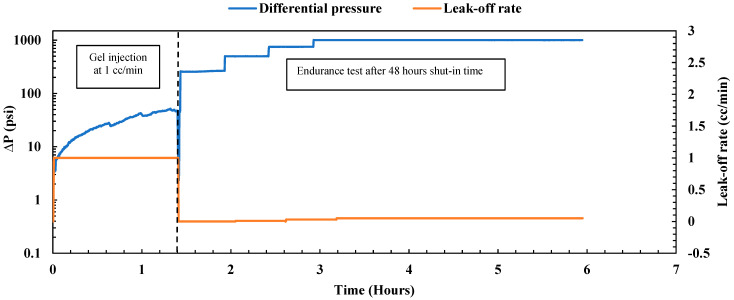
Nano-silica gel treatment on the induced channel sample.

Similarly, the experiment was conducted under reservoir conditions at 200 °F and an overburden pressure of 2000 psi. The sample’s permeability was obtained first by injecting brine at four different flow rates. It was found to be 440.60 mD, while the sample with a wormhole had a permeability of 5 mD. For gel injection, a gel concentration similar to that in experiment 1 was utilized; the prepared gel consisted of 75 wt% nano-silica and 25 wt% activator, providing a gelation time of 3 to 4 h. Figure 10 illustrates the observed pressure drop during gel injection at a rate of 1 cc/min, with continuous pressure observed due to the increasing viscosity of the solution with temperature (gelation). After 1.4 h, a total injected volume of 6.3 pore volumes was achieved, with the maximum pressure drop being less than 50 psi and a gel resistance factor of 47.2. This higher resistance factor compared to experiment 1 is attributed to the gel’s invasion into a low-permeability matrix surrounding the channel. The successful injection of the target volume with a low pressure drop indicates acceptable injectivity, ensuring the presence of the gel in both the matrix and channel.

Following the gel injection phase, the system underwent cleaning and was then sealed for 48 h to facilitate gel curing. Subsequently, the gel plugging performance was evaluated by the application of varying differential pressures as depicted in Figure 10. Initially, the sample was subjected to 250 psi for 30 min, followed by 500 psi for an additional 30 min, with no observed leak-off rate during either period, indicating efficient water flow blockage by the nano-silica gel in both channel and matrix areas. Droplets first appeared when applying 750 psi for 30 min, with a reported leak rate of 0.03 cc/min. To assess gel durability, the differential pressure was increased to 1000 psi and maintained for 3 h, during which the leak-off rate remained very low at 0.05 cc/min, with a holding pressure of 4000 psi/ft, highlighting the gel’s exceptional plugging capabilities under high differential pressure. The gel effectively obstructed both the channel path and the permeable matrix, achieving a plugging efficiency of 100%. Additionally, it demonstrated a high residual resistance factor of 90,802.96, significantly reducing the sample permeability from 440.60 mD to 0.0049 mD. These findings closely corroborate those of experiment 1.

**Figure 11:**
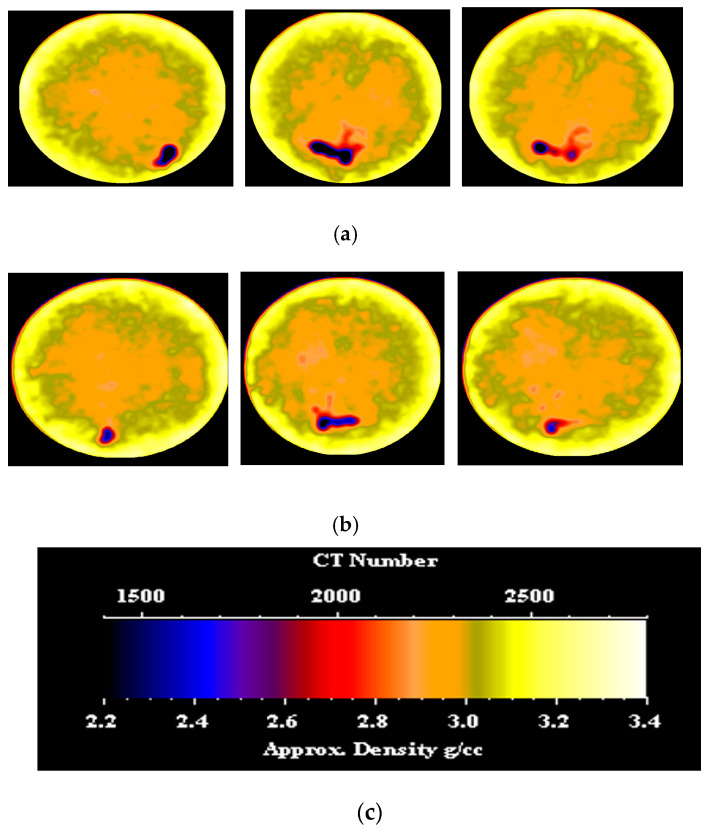
(**a**) CT scan of the induced channel before nano-silica gel treatment; (**b**) CT scan of the induced channel after nano-silica gel treatment; (**c**) color code scale.

CT scans were conducted on the core sample before and after nano-silica gel treatment. Figure 11 presents CT images of various cross-sections of both the untreated and treated samples. From the left side, the slices are located 0.5 inches, 1.7 inches, and 2.7 inches from the inlet face, respectively. Before the gel treatment, the channel was characterized by a circular spot with a dark color (low density). Following the application of the gel, a noticeable change in color to blue was observed (higher density). The presence of the gel changed the density distribution of the sample in the induced channel along the sample length. Before gel treatment, the average CT number of each slice was 2363. Post-treatment, this value significantly rose to approximately 2368.39. This marked increase confirms the successful placement and even distribution of the gel throughout the sample length in the induced channel and the surrounding matrix.

##### Gel Durability over a 6-Month Period

**Figure 12:**
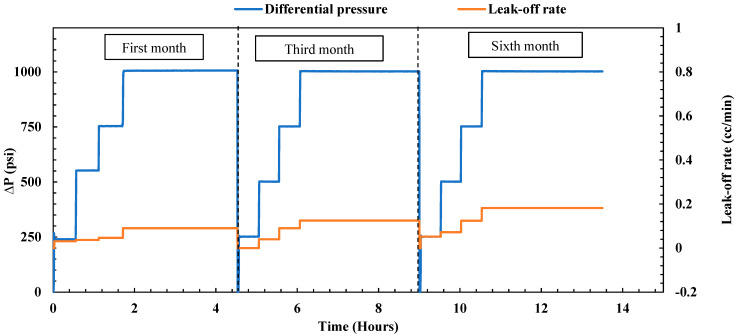
Nano-silica gel plugging efficiency over 6 months (induced channel sample).

**First month of aging:** The induced sample, having undergone treatment, was aged within a cell filled with formation water at a temperature of 167 °F for one month. Following this aging period, an endurance test was initiated by injecting brine at various differential pressures, as illustrated in Figure 12. The test began with a 30 min pressure hold at 250 psi, followed by another 30 min at 500 psi. During this hour, the leak-off rate was very minimal, measuring at 0.037 cc/min. Subsequently, the pressure was gradually increased to 750 psi for an additional half-hour, with the leak-off rate consistently remaining below 0.046 cc/min. To assess the durability of the gel, the pressure was maintained at a constant 1000 psi for 3 h, resulting in a reported leak-off rate of 0.09 cc/min. Although the maximum leak-off rate was slightly higher than the values observed in the post-treatment stage (0.05 cc/min), the reported leak-off rates were extremely low, indicating that the nano-silica gel maintained its plugging performance with a plugging efficiency of 100%.

**Third month of aging:** Following the initial month of endurance testing, the sample underwent further immersion in formation water at 167 °F for an additional two months. Subsequently, a subsequent endurance test was conducted at 200 °F. As depicted in Figure 12, a minor rise in the leak-off rate was noted in the third month compared to the first. Pressures of 250, 500, and 750 psi were sustained for 30 min each, with the maximum leak-off rate remaining minimal at 0.09 cc/min. To evaluate the gel’s endurance, the pressure was maintained at a constant 1000 psi for 3 h, yielding a reported leak-off rate of 0.125 cc/min. In a comparison of this third-month leak-off rate with the post-treatment values (as depicted in Figure 10), a slight increase was observed. However, the leak-off rate remained extremely low, with the overall plugging efficiency of the gel remaining at 100%, demonstrating its outstanding sealing capabilities.

**Sixth month of aging:** After the third-month endurance test, the sample underwent an additional three months of aging in formation water at 167 °F. The endurance test followed the same procedure, gradually increasing the differential pressure to 1000 psi at 200 °F. The gel’s performance after the sixth month is visually depicted in Figure 12. A slight increase was observed after the sixth month compared to the third month. At 500 psi for 30 min, the leak-off measured around 0.072 cc/min. This increased slightly to 0.124 cc/min at 750 psi. When maintained at 1000 psi for 3 h, the leak-off stabilized at 0.182 cc/min, higher than the rate obtained in the third month (0.125). Nevertheless, the maximum leak-off rate obtained is exceptionally low, confirming the gel’s remarkable effectiveness in sealing the induced channel sample. These findings after 6 months affirm that the nano-silica gel maintained its durability, achieving a plugging efficiency of 100%. The results obtained also indicate that the nano-silica gel performs similarly in both fractured and induced channel samples.

The endurance tests over six months clearly demonstrate the nano-silica gel’s exceptional durability and minimal degradation under high-pressure conditions. In the first month, the gel exhibited strong plugging performance with leak-off rates remaining extremely low, even at pressures up to 1000 psi, indicating excellent initial stability. By the third month, the gel showed only a slight increase in leak-off rates, suggesting that it maintained most of its integrity. The sixth-month results reveal a further, but still minimal, rise in leak-off rates, with the gel continuing to achieve a high plugging efficiency of 100%. These consistent results across different pressure conditions and aging periods underscore the gel’s robustness and effectiveness for long-term applications.

#### 2.2.2. Experiment 4 (PAM/PEI Gel on the Induced Channel Sample)

In this experiment, a limestone sample containing an induced channel was employed. The channel was sealed from one side to facilitate the invasion of the PAM/PEI gel into the matrix. Detailed information regarding the dimensions and properties of the sample can be found in the accompanying Table 4.

##### Gel Treatment

**Figure 13:**
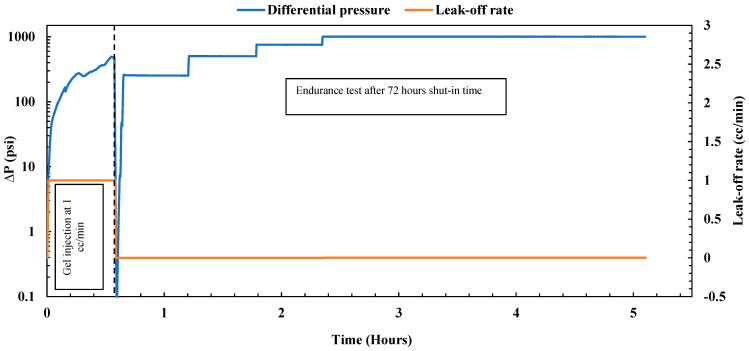
PAM/PEI gel treatment on the induced channel sample.

The experiment was conducted under reservoir conditions of 200 °F and a confining pressure of 2000 psi. Brine was injected into the saturated sample at four different flow rates. The sample’s permeability was found to be 10.48 mD. Since the channel was closed from the other side, the brine was forced to move through the matrix, resulting in the low reported permeability. For gel injection, the same concentration of PEM/PEI gel as in experiment 1 (9/1 wt%) was utilized. The injection process was finalized in approximately 30 min at 200 °F, as illustrated in Figure 13. The injection stage persisted for 30 min at a flow rate of 1 cc/min. The considerable pressure drop observed was attributed to the gel invasion into the low-permeability matrix, ensuring its placement in both the channel and the matrix. The total injected volume amounted to 3 times the pore volume.

Following the gel injection, the system underwent cleaning and then was sealed for a duration of 72 h to enable the gel to solidify at 200 °F. Subsequently, the endurance test was conducted by injecting brine under various differential pressures and monitoring the leak-off rate, as depicted in Figure 13. Initially, the sample endured 250 psi for 30 min, followed by an additional 30 min at 500 psi, without any observable leakage during either phase, indicating the initial high plugging efficiency of the nano-silica gel in sealing the induced channel sample. The pressure was then increased to 750 psi and sustained for 30 min, with no water droplets detected at the outlet. To evaluate the durability of the gel, the pressure was further raised to 1000 psi and maintained for 3 h. The gel effectively blocked both the channel path and the permeable matrix, exhibiting a minimal leak-off rate of 0.001 cc/min. The gel achieved a remarkable plugging efficiency of 100%, as it demonstrated a significant residual resistance factor of 771,082.4, resulting in a notable reduction in sample permeability from 10.48 mD to zero mD. These results confirm the gel’s ability to prevent water flow in channels.

**Figure 14:**
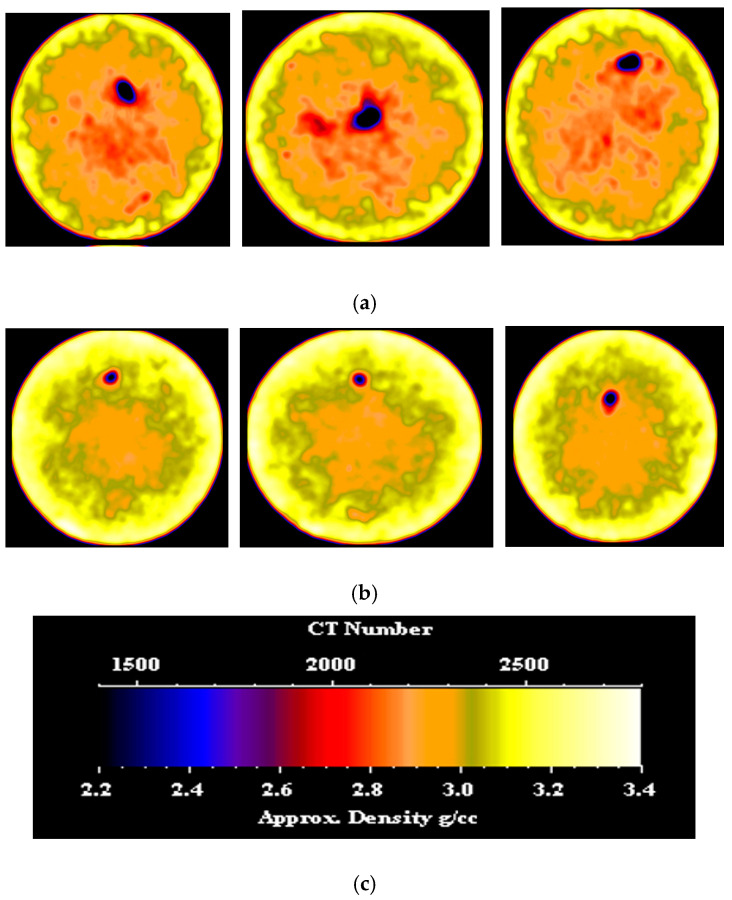
(**a**) CT scan of the induced channel before PAM/PEI gel treatment; (**b**) CT scan of the induced channel after PAM/PEI gel treatment; (**c**) Color code scale.

To visually assess the impact of gel invasion on pore space, CT scans were conducted before and after gel treatment. Figure 14 displays various slices of the core sample, with the induced channel appearing as a small dark spot in each slice, indicative of low density. From the left side, the slices are located 0.5 inches, 1.5 inches, and 2.7 inches from the inlet face, respectively. Following treatment, the color of these spots changed to blue, with a smaller size, suggesting a higher density distribution and confirming the presence of the gel within the channel. Furthermore, the average CT number of each slice along the induced sample length increased post-gel injection from 2305.12 to 2424.67, confirming the uniform distribution of the gel throughout the sample length.

##### Gel Durability over 6 Months

**Figure 15:**
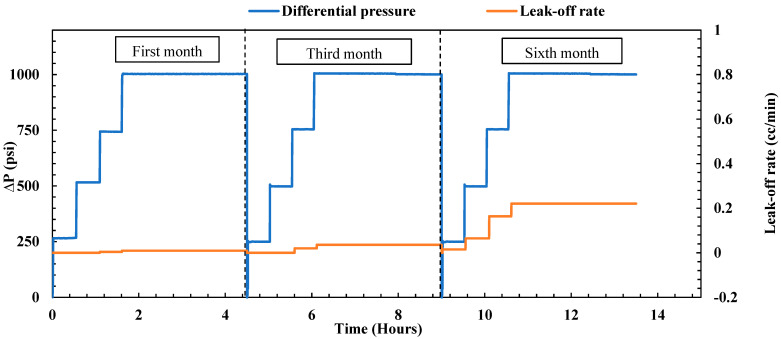
PAM/PEI gel performance over 6 months (induced channel sample).

**First month:** The sample endured a one-month aging period in formation water at 167 °F, followed by a 4.5 h endurance test at 200 °F, as depicted in Figure 15. The test commenced with a 30 min pressure hold at 250 psi, revealing no observable leak-off rate at the outlet. Subsequently, the pressure increased to 500 psi for 30 min and further to 750 psi for an additional half-hour. Throughout this period, the maximum leak-off rate remained below 0.004 cc/min, closely resembling the post-treatment level under the same differential pressure. To assess gel durability, a constant pressure of 1000 psi was sustained for 3 h, yielding a reported leak-off rate of 0.009 cc/min. Remarkably, the maximum leak-off rate remained consistent, indicating the gel’s stability at high differential pressure and sustained plugging efficiency at nearly 100%.

**Third month:** Following the first-month endurance test, the sample was replaced in formation water at 167 °F for an additional two months. Subsequently, another endurance test was conducted under varying differential pressures, reaching up to 1000 psi at 200 °F. As depicted in Figure 15, a slight increase in the leak-off rate was observed in the third month compared to the first month. Initially, the pressure was maintained at 250 psi for 30 min, during which no water droplets were observed. Subsequently, as the pressure was raised to 500 psi for 30 min, no leak-off rate was observed. The subsequent increase in pressure to 750 psi for 30 min led to a leak-off rate of 0.02 cc/min, indicating a minor increase. To assess the gel’s endurance, the pressure was sustained at a constant 1000 psi for 3 h, resulting in a reported leak-off rate of 0.036 cc/min. In a comparison of these leak-off rates after 3 months with the post-treatment values, as depicted in Figure 13, a slight increase was observed. However, this leak-off rate was extremely low, and the gel exhibited continued effectiveness, achieving an exceptional plugging efficiency of 99.99%.

**Sixth month:** Following the third-month endurance test, the sample underwent an additional three-month immersion in formation water at 167 °F. The endurance test was conducted similarly, with a gradual increase in the differential pressure up to 1000 psi at 200 °F. The gel’s performance during the sixth month is depicted in Figure 15, showing a slight resemblance in the leak-off rate compared to the third month, indicating minimal gel degradation. Under a pressure of 500 psi for 30 min, the leak-off rate was approximately 0.065 cc/min. This rate increased to 0.164 cc/min when the pressure was raised to 750 psi. Sustaining a pressure of 1000 psi for 3 h led to the leak-off rate stabilizing at 0.211 cc/min, notably higher than the rate in the third month (0.036). These findings suggest minimal gel degradation over the past 3 months. However, the PAM/PEI gel retained its plugging capabilities, achieving a plugging efficiency of 99.91% 6 months after the initial treatment.

The endurance tests of the PAM/PEI gel over six months, as shown in Figure 15, demonstrate its impressive long-term performance under high-pressure conditions. In the first month, the gel showed almost no leak-off, even at 1000 psi, indicating exceptional initial sealing capabilities and stability. By the third month, while there was a slight increase in leak-off rates, the values remained extremely low (0.036 cc/min at 1000 psi), with the gel maintaining a near-perfect plugging efficiency of 99.99%. After six months, the leak-off rate increased more noticeably, reaching 0.211 cc/min at 1000 psi, signaling some gel degradation. However, the gel still achieved a high plugging efficiency of 99.91%, reflecting its durability and reliability even 6 months after the initial treatment.

#### 2.2.3. Comparison of Gel Performance in the Induced Channel Samples

**Figure 16:**
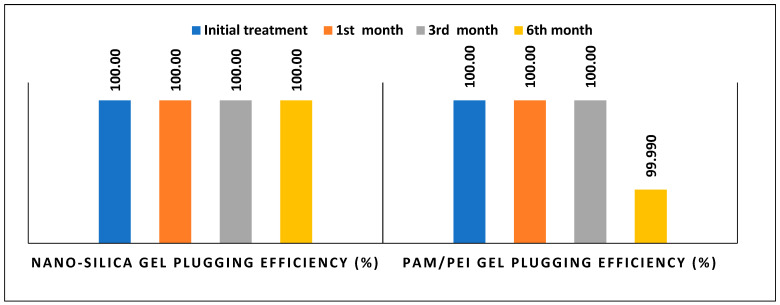
Comparison of plugging efficiency of nano-silica gel and PAM/PEI gel in the induced channel sample.

Two induced channel samples were treated with nano-silica gel and PAM/PEI gels, and their plugging efficiency over 6 months was compared, as shown in Figure 16. Both gels demonstrated sustained high plugging efficiency throughout the 6-month period. However, in the third month, nano-silica gel exhibited slightly better performance than PAM/PEI gel, which showed minor degradation affecting its performance in the last three months. Specifically, after 6 months, the leak-off rate for PAM/PEI gel was 0.221 cc/min, which was 1.2 times higher than that of nano-silica gel. This suggests that nano-silica gel may offer better long-term stability and effectiveness for water shut-off applications compared to PAM/PEI gel.

## 3. Conclusions

This study aimed to evaluate the long-term performance of nano-silica gel and to compare it with PAM/PEI gel as water shut-off agents in fractured and induced channel reservoir samples over a period of six months. The results demonstrated that nano-silica gel consistently maintained superior plugging efficiency compared to PM/PEI gel across both fracture and channel samples. This provides a significant improvement in the performance of silicate gels, which previously suffered from performance degradation over time. The key findings of the study include the following:

- Nano-silica gel maintained 100% plugging efficiency throughout the six months in both fractured and induced channel samples, even under high differential pressures (up to 1000 psi).
- PAM/PEI gel showed a slight decline in plugging efficiency, particularly in fractured samples, where it decreased to 99.15% after six months, but it retained 99.99% efficiency in the induced channel samples.
- Nano-silica gel proved to be a more durable and stable option for long-term water shut-off applications compared to PAM/PEI gel.

## 4. Materials and Experimental Plan

### 4.1. Chemicals

**Figure 17:**
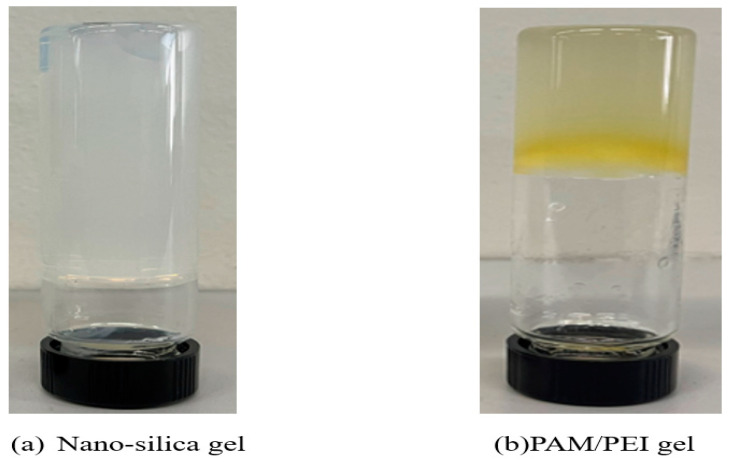
Gel samples after curing: (**a**) nano-silica gel; (**b**) PAM/PEI gel.

Two gel systems were utilized in this study: nano-silica gel and PAM/PEI gel. Nano-silica gel was composed of a mixture of nano-silica solution and activator, both provided as bulk fluid by (Saudi Aramco, Dhahran, Eastern province, Saudi Arabia), mixed in specific weight proportions to ensure sufficient gelation time for optimal placement. The nano-silica solution consisted of 40% wt nano-silica as the active material, with the remainder being water. Also, the activator’s composition was approximately 30% by weight sodium chloride, with the remainder being water. Regarding stability, the nano-silica solution is considered highly thermally stable since its primary component is silica. According to the manufacturer, the nano-silica solution remains stable at temperatures up to 350 °F. To prepare a 75/25 wt% nano-silica gel, a predetermined volume of the silica solution was measured in a 500 mL beaker, and its weight was determined, followed by the addition of an equivalent volume of activator based on the desired ratio at atmospheric conditions. The mixture was stirred for 10 min until a uniform consistency suitable for injection was achieved. The silica nanoparticles exhibited a discrete, smooth, spherical shape, with an average particle size distribution of 14 nm as analyzed by the FPIA-3000 (Malvern) particle size analyzer. Initially, the gel mixture presented an average viscosity of 6 centipoises (cp), which increases with temperature due to gelation. A cured sample of nano-silica gel after 24 h is displayed in Figure 17.

The second gel system, referred to as PAM/PEI gel, comprised two primary solutions: PAM (polyacrylamide) and PEI (polyethyleneimine). PAM, obtained from SNF Floerger in the form of an aqueous solution, was utilized without further treatment, featuring a concentration of 20 wt% active ingredients and a molecular weight ranging from 250 to 500 kg/mol, as specified by the supplier. PEI, serving as a cross-linker, was provided in solution form. To prepare 30 mL of 9/1 wt% PAM/PEI gel, 13.5 mL of PAM was initially combined with 15.5 mL of water and stirred for approximately one minute. Following this, 1 mL of PEI was introduced to the mixture, which was then stirred for an additional 10 min. Figure 17 shows a PAM/PEI gel sample that was fully cured after 72 h. For measuring permeability and conducting endurance tests, a brine solution containing sodium chloride, mainly at a concentration of 0.1 M per liter, was utilized.

### 4.2. Methodology

#### 4.2.1. Experimental Plan

**Figure 18:**
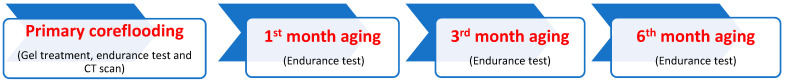
Stages of gel performance evaluation.

The study plan involved initially treating fractured core samples with gel, followed by an endurance test to verify the initial plugging efficiency. The endurance test was designed to evaluate the treated core sample’s ability to resist water flow under differential pressures ranging from 250 psi to 1000 psi over a duration of 4.5 h. Subsequently, the gel-plugged sample underwent three aging stages in formation water over six months. A detailed experimental plan for this duration is outlined in Figure 18. After the first month of aging, an endurance test was conducted to reevaluate the gel plugging efficiency. These procedures were repeated after the third and sixth months of aging. After the sixth month, the gel’s plugging performance was evaluated and compared to its initial effectiveness while also determining the degradation rate. This systematic approach provided valuable insights into the long-term efficiency and stability of the gel.

#### 4.2.2. Coreflooding System

**Figure 19:**
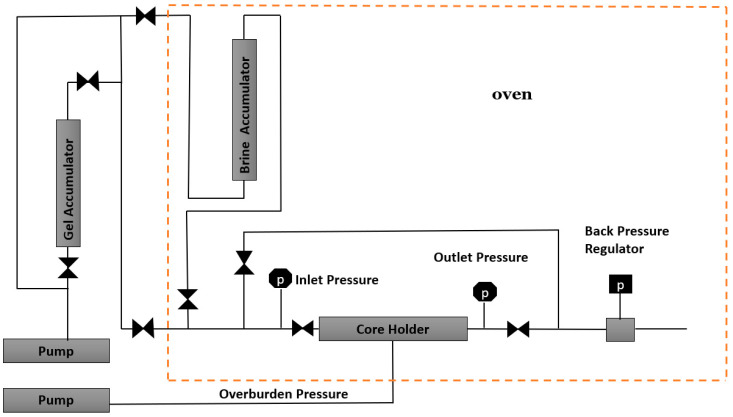
Coreflooding equipment for gel treatment and endurance test.

A fully equipped coreflooding system was utilized to conduct the initial gel treatment and endurance test on the fractured sample. Figure 19 shows the main components of the coreflooding system. This system includes a 1.5-inch core holder fitted with two pressure transducers to measure inlet and outlet pressures accurately. Additionally, it features dual pumps: one for applying confining pressure to the core holder and the other for injecting fluid via two accumulators—one outside the oven for gel and another inside for brine. To maintain pressure consistency and replicate reservoir conditions, a back pressure regulator is employed, set at 400 psi. Moreover, medical CT scans were utilized to characterize the density distribution of the samples before treatment, after treatment, and during the aging stages.

To ensure effective gel treatment, a sufficient amount of gel was injected into the brine-saturated sample at a very low rate of 1 cc/min for 1.5 to 2 h, aligned with the gelation time of the injected gel. Subsequently, the system underwent a thorough cleaning to remove any remaining gel residue before being shut in for 48 to 72 h to allow the complete curing of the gel. Once the gel was fully cured, the endurance test commenced, and the sample was subjected to various differential pressures at 200 °F (2000 psi confining pressure) while the leak-off rates were monitored. The values of the differential pressure and the leak-off were used to evaluate the permeability at each stage using Darcy’s law. The leak-off rate with differential pressure can be used to estimate the sample’s permeability after treatment. Plugging efficiency was calculated using Equation (1):

(1) $$Plugging\;efficiency\;(\%)=\frac{Initial\;Permeability-Final\;Permeability}{Initial\;Permeability}\;\times \;100$$

#### 4.2.3. CT Scan

To ensure optimal plugging efficiency with the gel, it is crucial to have a uniform distribution of an adequate amount of gel throughout the sample. In the experiments that were conducted, a medical CT scan was utilized to identify the distribution pattern and penetration length of the gel precisely along the sample length. CT scans were conducted before treatment and after treatment. The results were obtained in terms of CT numbers, which represent the intensity of the transmitted X-ray beam and correspond to specific density values in g/cm^3^ [35]. Therefore, variations in CT numbers directly indicate changes in density, reflecting the extent of gel invasion.

## Figures and Tables

**Table 1:** Core sample dimensions and properties for experiment 1.

Length (Inches)	Diameter (Inches)	Porosity (%)
3.0	1.5	21.88

**Table 2:** Core sample dimensions and properties for experiment 2.

Length (Inches)	Diameter (Inches)	Porosity (%)
3.0	1.5	20.54

**Table 3:** Core sample dimensions and properties for the core sample of experiment 3.

Length (Inches)	Diameter (Inches)	Porosity (%)
3.0	1.5	15.70

**Table 4:** Core sample dimensions for the core sample of experiment 4.

Length (Inches)	Diameter (Inches)	Porosity (%)
3.0	1.5	13.04

## Data Availability

No external data were used for this research. All the generated experimental data are included in this manuscript. The datasets used and/or analyzed during the current study are available from the corresponding author on reasonable request.
